# Case Report: Beware of arteria lusoria: a potential risk factor in esophagectomy surgery

**DOI:** 10.3389/fsurg.2025.1548041

**Published:** 2025-03-12

**Authors:** Quanrui Liu

**Affiliations:** Department of Thoracic Surgery, Nanbu County People's Hospital, Nanchong City, Sichuan, China

**Keywords:** esophagectomy surgery, computed tomography, arteria lusoria, vascular anomalies, arterial injury

## Abstract

The aberrant right subclavian artery (ARSA), also known as lusoria artery, is a relatively rare vascular malformation influencing 0.6% to 1.4% of the population, and this figure elevates exponentially to 26%–34% among Down-syndrome individuals. However, few cases are available on esophageal cancer associated with ARSA. Here, we report a 30-year-old male esophageal cancer patient with arteria lusoria, which elevated surgical risks in esophagectomy. We reviewed the key points of anatomy and further complications to understand this vascular anomaly in esophagectomy. This report aims to raise awareness among thoracic surgeons and radiologists about the importance of thorough preoperative assessment and the unique challenges posed by ARSA in esophageal cancer surgery.

## Introduction

The aberrant right subclavian artery (ARSA), also known as lusoria artery, is a rare congenital vascular anomaly that arises from the descending aorta rather than from the brachiocephalic trunk, as is typical for most individuals. This anomaly occurs in approximately 0.6%–1.4% of the population but is much more common in individuals with Down syndrome, with an incidence rate between 26% and 34% (1). Although ARSA is often asymptomatic, its presence becomes clinically relevant in surgeries involving the thoracic cavity, especially those involving the esophagus or the aortic arch.

In esophagectomy procedures, careful preoperative assessment and surgical planning are essential, as the vascular anomaly can lead to intraoperative complications, including arterial injury and esophageal-arterial fistula (2). However, the clinical significance of ARSA in esophageal cancer surgery has not been extensively documented. Here, we describe a case of a 30-year-old male patient with esophageal SCC, who was found to have ARSA, and how this anomaly influenced the surgical approach. This case highlights the complexity of managing patients with both esophageal cancer and ARSA, providing insights into the critical role of preoperative imaging and careful surgical planning to avoid complications. By providing a detailed account of this case, we aim to increase awareness of ARSA among thoracic surgeons and radiologists.

## Case presentation

**Figure 1:**
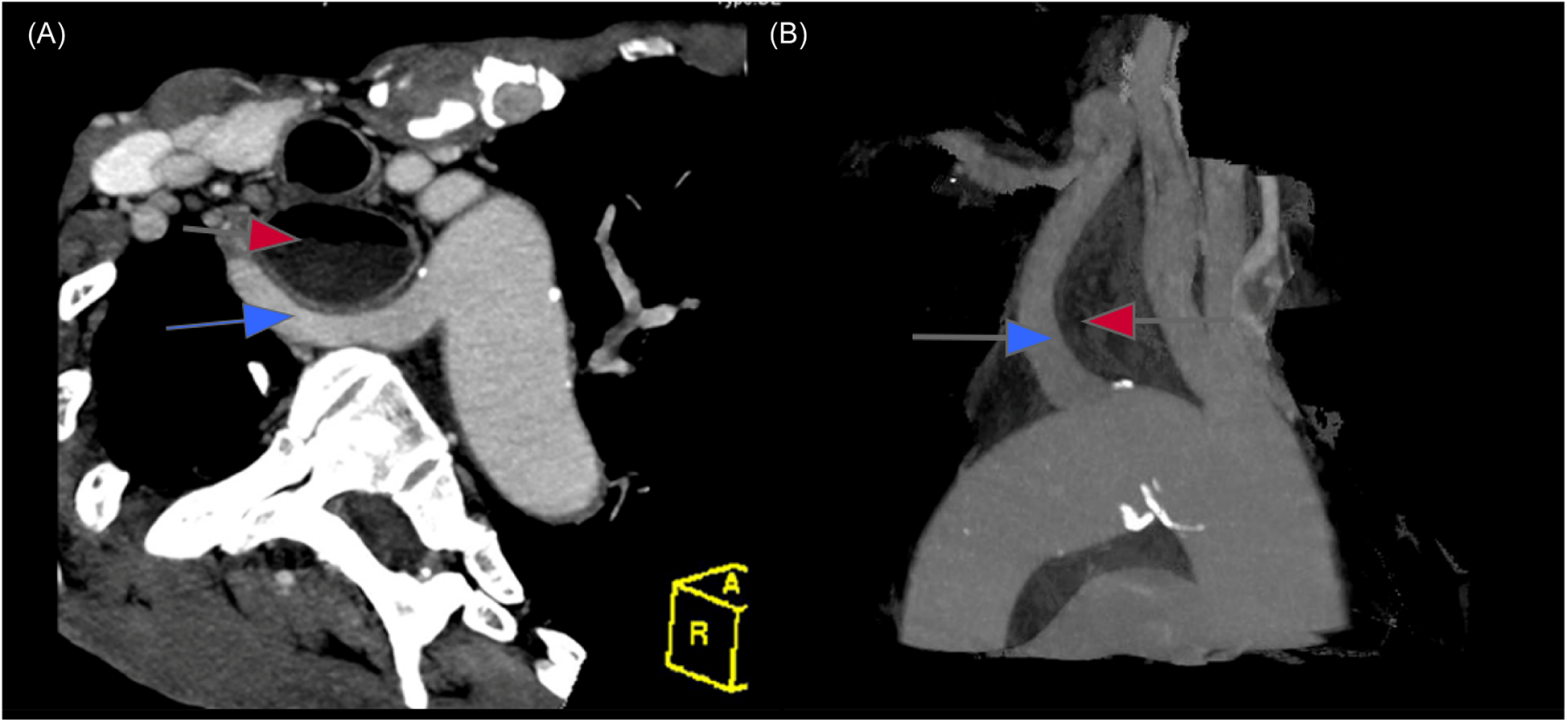
Computed tomography (axial reformation **(A)** and sagittal reformation **(B)** showed an ARSA (blue arrow) extends from the left side of the aortic arch and running bhind the esophagus (red arrow).

**Figure 2:**
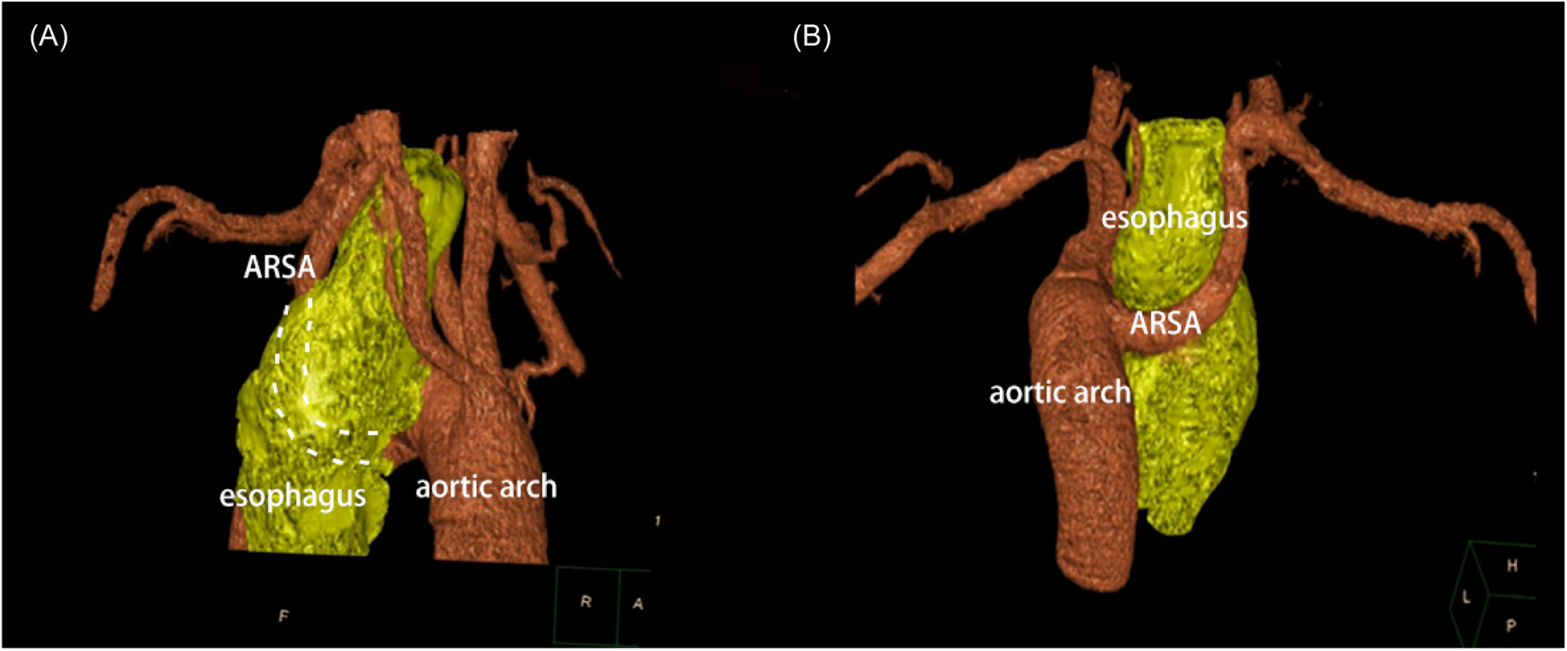
Three-dimensional reconstruction imaging (anteroposterior and posteroanterior view) presented that ARSA traveling retroesophageal before supplying the upper limb.

A 30-year-old male patient was referred to our hospital with progressive dysphagia and weight loss over the past few months. Upper endoscopy revealed a large ulcerative lesion in the mid to distal thoracic esophagus. A biopsy of the mass was taken, and histological analysis confirmed the presence of moderately differentiated squamous cell carcinoma (SCC). Preoperative staging, including computed tomography (CT) and positron emission tomography (PET) scans, demonstrated a locally advanced esophageal cancer without distant metastasis, corresponding to stage IIIB (T3N2M0) esophageal SCC. Interestingly, imaging also revealed a rare vascular anomaly: a lusoria artery (Figures 1A,B). This anatomical variation involves the right subclavian artery arising from the descending aorta and crossing posterior to the esophagus to supply the right upper limb (Figures 2A,B). The clinical challenge of diagnosing both esophageal cancer and arteria lusoria was significant, as the symptoms of dysphagia and weight loss could easily be attributed solely to the malignancy, making the identification of the vascular anomaly more difficult. The presence of this abnormal artery added complexity to the surgical planning, requiring meticulous preoperative assessment.

**Figure 3:**
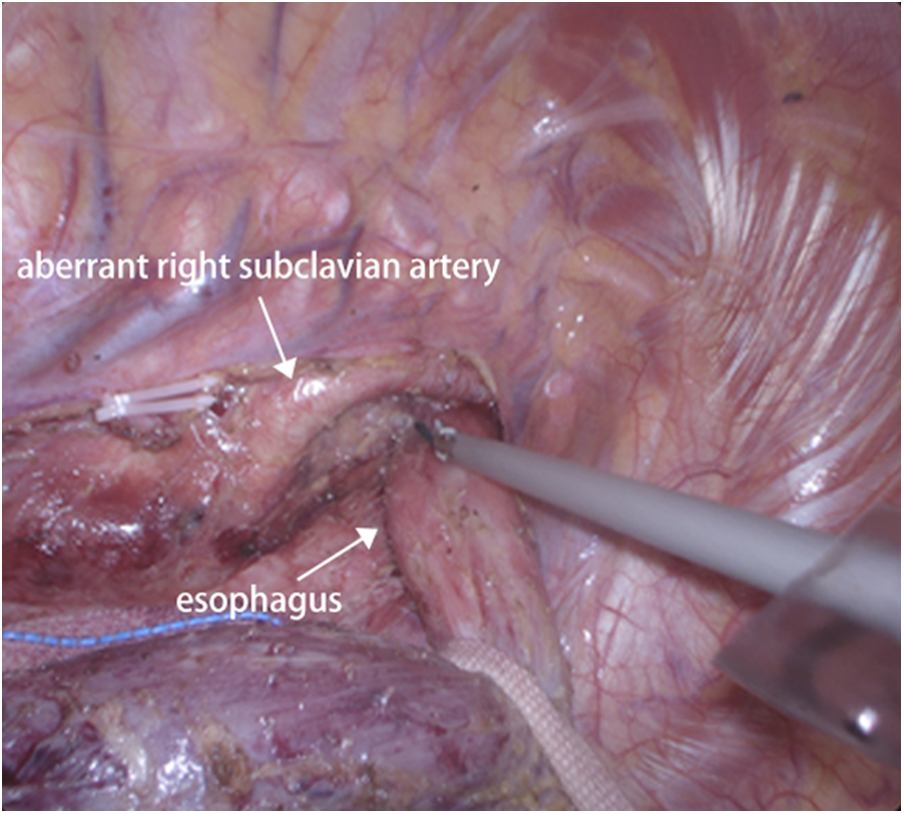
Intraoperatively view during thoracoscopic esophagectomy presented an ARSA crossing the esophagus posteriorly.

Given the patient's advanced stage of esophageal cancer, we initiated a neoadjuvant chemoimmunotherapy regimen comprising cisplatin and fluorouracil, combined with pembrolizumab. The patient tolerated the neoadjuvant therapy well, with no severe adverse events, and showed a partial response on imaging after completing the chemotherapy course. Following neoadjuvant therapy, the patient underwent a minimally invasive subtotal esophagectomy with extended lymphadenectomy and esophagogastric anastomosis via video-assisted thoracoscopic surgery (VATS). During surgery, careful dissection in the mediastinum revealed the lusoria artery, which was confirmed visually (Figures 3). The identification of the arteria lusoria in the preoperative imaging allowed for a more precise and controlled dissection during surgery, minimizing the risk of vascular injury. The course of the right recurrent laryngeal nerve (RLN) was also carefully monitored throughout the procedure, as ARSA is known to be associated with variations in the RLN anatomy. In this case, the right RLN was found to take a more posterior and inferior position due to the presence of the aberrant artery. Intraoperative nerve monitoring was used to ensure the nerve was not damaged during dissection. The operation was completed without intraoperative complications and was discharged on the 14th postoperative day in stable condition, without any major complications such as anastomotic leakage, recurrent laryngeal nerve palsy, or chylothorax. A follow-up endoscopy three months after surgery showed no evidence of disease recurrence, and the patient remains on regular surveillance.

## Discussion

ARSA is a rare but clinically significant vascular anomaly that may pose substantial risks during thoracic surgeries, particularly esophagectomy. The anatomical variation results from abnormal development of the pharyngeal arch arteries during fetal development. ARSA typically arises from the descending aorta, which leads to its path posterior to the esophagus. Although most patients with ARSA are asymptomatic, its presence can complicate thoracic surgeries by increasing the risk of inadvertent vascular injury and other postoperative complications such as esophageal-arterial fistula or nerve damage (3).

In this case, the patient presented with esophageal squamous cell carcinoma, and preoperative imaging revealed ARSA. The use of high-resolution 64-slice CT imaging and 3D reconstruction allowed for accurate preoperative assessment of the ARSA's course, providing critical information that influenced surgical planning. The clinical difficulty in assessing both the esophageal lesion and the vascular anomaly highlights the importance of advanced imaging techniques, such as multislice CT with 3D reconstruction, to avoid misdiagnosis or complications. Multislice CT imaging offers 100% diagnostic sensitivity for identifying ARSA (4), making it a valuable tool for preoperative evaluation. In this patient, the 3D reconstructed CT images provided comprehensive visualization of the aorta and its major branches, helping to map out the anatomy and avoid potential complications during surgery.

One of the most significant concerns when performing esophagectomy in patients with ARSA is the potential for arterial injury, which can lead to severe bleeding (5). Blunt dissection techniques, commonly used in esophageal surgeries, should be avoided in these cases due to the risk of damaging the abnormal artery. It is essential to perform careful and controlled dissection, particularly when the ARSA is located in close proximity to the esophagus. In some cases, open thoracotomy may be considered if complications arise, as it provides greater exposure and control over the surgical site.

Another critical consideration is the potential for injury to the non-recurrent inferior laryngeal nerve (NRILN), which is a variant that may be more susceptible to damage when ARSA is present. Intraoperative monitoring of the laryngeal nerve is highly recommended to prevent nerve injury during dissection (6). In addition, careful neck dissection may be required before performing VATS in order to identify and manage the ARSA and other vascular anomalies safely. Moreover, the rare occurrence of esophageal-arterial fistula following esophagectomy, though low in incidence, is a severe complication that can result from vascular injury, leading to potentially fatal outcomes (7, 8). If postoperative hematemesis or hypotension occurs, immediate investigation for this complication is necessary, particularly when the patient has known vascular anomalies like ARSA (9).

## Conclusion

Arteria lusoria is a rare but significant anatomical variant that can complicate esophagectomy surgery. Preoperative identification and careful surgical planning are critical to avoid catastrophic complications such as arterial injury and esophageal-arterial fistula. The case highlights the importance of preoperative imaging and the need for careful surgical planning in patients with both esophageal cancer and arteria lusoria. Surgeons should remain vigilant when encountering patients with ARSA and consider advanced imaging techniques to fully understand the vascular anatomy. By raising awareness of this potential risk factor, we hope to improve patient outcomes in esophageal cancer surgery.

## Data Availability

The raw data supporting the conclusions of this article will be made available by the authors, without undue reservation.
